# Cancerogenic parasites in veterinary medicine: a narrative literature review

**DOI:** 10.1186/s13027-023-00522-x

**Published:** 2023-07-26

**Authors:** Niccolò Fonti, Francesca Parisi, Francesca Mancianti, Giulia Freer, Alessandro Poli

**Affiliations:** 1 Dipartimento di Scienze veterinarie, Università di Pisa, Viale delle Piagge, 2, 56124 Pisa, Italy; 2 Dipartimento di Ricerca Traslazionale e delle Nuove Tecnologie in Medicina e Chirurgia, Università di Pisa, Via Savi, 10, 56126 Pisa, Italy

**Keywords:** Cancer, Parasites, Veterinary oncology, *Spirocerca*, Flukes, *Theileria*, *Heterakis*, Helminths, Host-parasite relationship

## Abstract

Parasite infection is one of the many environmental factors that can significantly contribute to carcinogenesis and is already known to be associated with a variety of malignancies in both human and veterinary medicine. However, the actual number of cancerogenic parasites and their relationship to tumor development is far from being fully understood, especially in veterinary medicine. Thus, the aim of this review is to investigate parasite-related cancers in domestic and wild animals and their burden in veterinary oncology. Spontaneous neoplasia with ascertained or putative parasite etiology in domestic and wild animals will be reviewed, and the multifarious mechanisms of protozoan and metazoan cancer induction will be discussed.

## Introduction

With an estimated 19.3 million new cases of cancer and almost 10 million deaths in 2020, cancer is a major threat to public health globally [1]. Many environmental variables, such as pathogens and unhealthy lifestyles, can contribute significantly to carcinogenesis. Infectious diseases account for 15.4% of the worldwide cancer burden [2], reaching 20% in developing countries [3]. Indeed, it has been predicted that infections will account for most human cancer cases by 2050 [4].

There are 11 species of pathogenic microorganisms that are "carcinogenic to humans" and categorized as group 1 carcinogens according to the International Agency for Research on Cancer (IARC) [5]. Among these, *Helicobacter pylori* and viruses such as the human papillomavirus (HPV), hepatitis B virus (HBV), hepatitis C virus (HCV), and Epstein-Barr virus (EBV) are well-known biological carcinogens [1, 5]. Three helminthic species are also included in group 1: *Opistorchis viverrini*, *Clonorchis sinensis*, and *Schistosoma haematobium* [3, 5–7]. In addition, *Schistosoma japonicum* has been assigned to group 2B (possible carcinogens), and *Schistosoma manson*i and *Opisthorchis felineus* to group 3 (not classifiable carcinogens) [8, 9]. Furthermore, the previously neglected notion that other metazoan and protozoan parasites may also contribute to malignancy development is now being evaluated [10, 11].

The oncologic burden is increasing in veterinary medicine as well [12], and the list of pathogens responsible for animal cancer development continues to grow [13, 14]. In addition to various viral [15], putative bacterial species [16], and transmissible tumor cells [17–19] that cause cancer, certain parasites have also been identified over the years, although an overlooking of their impact on animal health was suggested [20–24]. While breakthroughs in the field of parasite-related carcinogenesis have mainly concerned humans so far, understanding the role of these parasites in veterinary medicine could lead to significant progress in prevention and new potential therapies for animal species as well [13]. Furthermore, a deeper knowledge of this phenomenon would benefit human medicine in a variety of ways. Wild and domestic animals have always provided unique models to elucidate the infectious-driven neoplastic phenomena, and their use in modern oncology is increasing [25–27]. The high occurrence of parasitic infection and the wider biodiversity in host-parasite interactions in the veterinary field compared to humans, as well as the generally lower awareness of their zoonotic potential (compared to viruses and bacteria), are all significant factors in opening new and unpredictable perspectives for research on cancerogenic parasites [25, 26, 28]. Still, the literature on cancer-causing parasites in the veterinary field is extremely fragmented, with some pathogens well-characterized (such as *Spirocerca lupi*) [22], and others that are ignored or only known anecdotally. While cancerogenic parasites in humans are widely investigated and updated reviews are provided [7, 29–35], there is a gap in the veterinary field and parasites of veterinary importance are often not included in these discussions.

Therefore, the aim of this paper is to comprehensively review the current literature on cancer-causing parasites in veterinary medicine, gathering articles on naturally occurring parasite-induced cancer in wild and domestic animals in a comprehensive way and providing an overview of the cancerogenic mechanisms involved.

## Materials and methods

The literature search on PubMed and Google Scholar used the terms "parasite OR helminths OR protozoa" AND "cancer OR neoplasia OR tumor OR malignancy," and various combinations of these terms were employed. A second specific search was based on the results of this first literature search and restricted to parasitic agents in which an etiopathogenetic role was assumed or ascertained in spontaneous malignancies in wild and domestic animals. The search was conducted using the scientific names of parasite species (*Gongylonema* spp.; *Heterakis* spp.; *S. lupi*; *Ophidascaris* spp.; *Ollulanus trichuspis*; *Nochtia nochti*; *Trichinella* spp.; *C. sinensis*; *Opistorchis* spp*.*; *Fasciola* spp., *Platynosomum* sp.; *Schistosoma* spp., *Taenia* spp*.*, *Linguatula serrata*, *Theileria* spp.) or their taxa (e.g., Trematoda; Cestoda; Platyhelminthes, Nematoda; Apicomplexa; Arthropoda) combined with the names of the specific malignancy and related host species. Observational studies such as case reports, case series, cross-sectional, and case–control studies were included. There was no set time restriction, and additional literature was found using a reverse and forward snowball search strategy. Parasites such as *Gongylonema neoplasticum, O. felineus*, *Cryptosporidium* spp., whose carcinogenic role has not been demonstrated but cannot be ruled out based on in vitro or in vivo experimental research, were also mentioned in the discussion.

In the first part of the review, a brief overview on the etiology, life cycle, and zoonotic potential of each parasite is conducted before discussing the neoplastic lesions associated. In the second part, an outline of ascertained and putative cancerogenic mechanisms is provided.

In vivo studies concerning parasite-induced cancer in experimental animals and in vitro studies were excluded. However, the results of experimental studies focusing on the cancerogenesis mechanisms were mentioned in the second part of the paper. The search methodology adheres to the recommended standards for effective narrative review articles [36, 37].

## Parasites associated with neoplasia in domestic and wild animals

The original articles retrieved in the literature search that focused on the causal association of parasites with spontaneous neoplasia in veterinary medicine are summarized in Table 1. Scientific papers on *S. lupi* are not included in Table 1 for readability reasons, but they will be discussed in the corresponding section. The parasite-cancer relationship, both putative and confirmed, was observed in a range of domestic (dog, cats, ruminants, rats, mice, and poultry species) and wild or exotic species (prosimians, New-World and Old-World monkeys, snakes, and muskrats). A total of 15 parasite genera were identified, with the majority (14) being metazoan, especially (but not exclusively) helminths. The parasitic species identified were trematodes (*C. sinensis*, *Fasciola* spp., *O. viverrini*, *Platynosomum illiciens*, *S. mansoni*), cestodes (*T. taeniformis*), nematodes (*G. pulchrum*, *S. lupi*, *Heterakis* spp., *Nochtia nochti, Ollulanus trichuspis*, *Ophidascaris* sp., *Trichinella* spp.), and arthropods, such as pentastomides (*Linguatula serrata*). An additional protozoan parasite was identified (*T. annulata*). This trend mirrors that of human medicine, where helminths initially received most of the attention [29, 30], but more recent improvements in diagnostic tools have shifted emphasis to other putative parasitic agents, including protozoa [11, 38, 39].

**Table 1:** Spontaneous parasite-related neoplasia reports in wild and domestic animals

Phylum	Parasite species	Host species	Associated neoplasms	Cases	References
Nematoda	*Gongylonema pulchrum*	*Lemur macaco variegata*	esophageal SCC	1	[67]
	*Heterakis gallinarum*	*Phasianus colchicus*	leiomyoma	8	[124]
	*Heterakis gallinarum*	*Phasianus versicolus*	fibrosarcoma	1	[132]
	*Heterakis isolonche*	*Phasianus colchicus*	mesenchymal tumor NOS	1	[133]
	*Heterakis isolonche*	*Crysolophus pictus*	leiomyoma	1	[134]
	*Heterakis isolonche*	*Crossoptilon auritum*	leiomyoma	2	[136]
	*Heterakis isolonche*	*Crossoptilon mantchuricum*	leiomyoma	1	[136]
	*Heterakis isolonche*	*Syrmaticus soemmerringii*	leiomyoma	1	[136]
	*Heterakis* sp*.*	*Crysolophus pictus*	leiomyoma	3	[135]
	*Ophidascaris* sp*.*	*Morelia spilota spilota*	gastric adenocarcinoma	1	[137]
	*Ollulanus trichuspis*	*Felis catus*	gastric adenocarcinoma	2	[144]
	*Nochtia nochti*	*Macacus mordax*	invasive gastric papilloma	6	[153]
	*Trichinella spiralis*	*Felis catus*	oral SCC	1	[170]
	*Trichinella* sp*.*	*Canis lupus familiaris*	melanoma	1	[169]
	*Clonorchis sinensis*	*Felis catus*	cholangiocarcinoma	2	[191]
Platyhelminthes	*Clonorchis sinensis*	*Canis lupus familiaris*	cholangiocarcinoma	1	[193]
	*Opistorchis viverrini*	*Felis catus*	biliary cystadenoma	1	[198]
	*Fasciola gigantica*	*Bos taurus*	leiomyoma	10	[216]
	*Fasciola gigantica*	*Bos taurus*	leiomyoma	44	[218]
	*Fasciola hepatica*	*Bos taurus*	HCC	11	[219]
	*Fasciola* sp*.*	*Bos taurus*	leiomyoma	1	[217]
	*Platynosomum illiciens*	*Felis catus*	cholangiocarcinoma	4	[227]
	*Platynosomum illiciens*	*Felis catus*	cholangiocarcinoma	3	[228]
	*Platynosomum* sp.	*Callithrix* sp*.*	HCC	1	[229]
	*Schistosoma mansoni*	*Pan troglodytes*	HCC	1	[232]
	*Taenia taeniformis*	*Rattus norvegicus*	renal sarcoma	1	[52]
	*Taenia taeniformis*	*Rattus norvegicus*	hepatic sarcoma	1	[52]
	*Taenia taeniformis*	*Rattus norvegicus*	hepatic sarcoma	11	[241]
	*Taenia taeniformis*	*Rattus norvegicus*	hepatic fibroma	1	[241]
	*Taenia taeniformis*	*Rattus norvegicus*	hepatic sarcoma	3	[242]
	*Taenia taeniformis*	*Rattus norvegicus*	hepatic fibrosarcoma	16	[240]
	*Taenia taeniformis*	*Rattus norvegicus*	hepatic fibrosarcoma	1	[244]
	*Taenia taeniformis*	*Rattus norvegicus*	hepatic fibrosarcoma	2	[245]
	*Taenia taeniformis*	*Rattus norvegicus*	hepatic sarcoma	1	[247]
	*Taenia taeniformis*	*Rattus norvegicus*	hepatic fibrosarcoma	48	[248]
	*Taenia taeniformis*	*Rattus norvegicus*	hepatic fibrosarcoma	ND	[249]
	*Taenia taeniformis*	*Rattus norvegicus*	hepatic fibroma	1	[250]
	*Taenia taeniformis*	*Mus musculus*	hepatic fibrosarcoma	7	[246]
	*Taenia taeniformis*	*Ondatra zibethicus*	hepatic fibrosarcoma	1	[243]
Arthropoda	*Linguatula serrata*	*Canis lupus familiaris*	nasal basosquamous carcinoma	1	[274]
Apicomplexa	*Theileria annulata*	*Bos taurus*	lymphoma	1	[288]

Phylum, parasite species, host species, neoplasm, and the total number of cases involved are reported. HCC, hepatocellular carcinoma; SCC, squamous cell carcinoma; ND, not determined. *Spirocerca lupi* is not included in this table

From a pathological standpoint, both mesenchymal and epithelial neoplasms were reported, with only one hematopoietic lesion (lymphoma).

### Some preliminary reflections: the jumping the gun of *Gongylonema neoplasticum*.

Theories suggesting a link between parasite diseases and neoplastic phenomena are very old and have been hypothesized for centuries [24, 40, 41]. However, several of these theories are no longer scientifically relevant, such as Sennert's (1572–1637) research on common leprosy and carcinoma etiology, and Justammond's (1737–1786) theory that cancer was caused by insects absorbed by lymphatic vessels [40, 41]. The scientific foundation of what is currently known about the association between cancer and infectious agents was established at the beginning of the twentieth century [42]. The discovery of the parasite *G. neoplasticum* is undoubtedly a remarkable and controversial story [43]. *Gongylonema* is currently a genus of widespread spirurid nematodes in the family Gongylonematidae, and humans are thought to be accidental hosts [44–46]. The squamous epithelial surface of the mouth, esophagus, rumen, and stomach of several birds and mammals are the locations where the adults reside and reproduce [47].

In 1907, when three wild rats were dissected, Danish physician Johannes Fibiger observed gastric tumors as well as roundworms inside them. This discovery prompted Fibiger's research, which identified the worm as a new species, *Spiroptera* (*Gongylonema*) *neoplastica*, and demonstrated that cockroaches were its intermediate hosts [48, 49].

The most significant finding in Fibiger’s work was the identification of a causal relationship between parasites and cancer by inducing the same tumors in rats and mice fed with *G. neoplastica* third larval stages (L3)-containing cockroaches. Fibiger also emphasized that the lesions were not merely papillomas or granulomas caused by foreign-body irritation, like other frequent worm-induced epithelial hyperplastic lesions. Instead, he accurately described squamous-cell carcinomas invading nearby tissues, with metastatic spread to many organs [50]. Parasitic worms had previously been implicated in cancerogenesis, as shown by *S. haematobium* and bladder cancer in humans in 1911 [51], as well as *T.* (*Hydatigera*) *taeniformis* and liver sarcoma in rats by Amédée Borrel in 1906 [52]. However, a technique for inducing cancer in lab animals was lacking; in this regard, Fibiger’s findings were astounding and gave rise to an impulse in cancer research [24], leading Fibiger to receive the Nobel Prize Award in Medicine and Physiology in 1926. The peculiar thing about this story is that Fibiger was wrong: a series of experiments carried out by different authors [53], culminating in the study written by Hitchcock and Bell [54], challenged the Danish Physiologist's findings. The results by Hitchcock and Bell showed that the worms were not the causative agents of the lesions, but that the "neoplasms" were mostly caused by a nutritional deficiency. Indeed, Fibiger's animals were subjected to a diet lacking in vitamin A, whereas the worms induced only mild hyperplasia and hyperkeratosis in rats that were well-fed [54]. Furthermore, the *Gongylonema-*associated growths were actually "hyperplastic hyperkeratotic papillomas" and the presumed metastases were patches of metaplastic tissue. Therefore, the beginning of the cancer-causing parasite investigations was a jump-the-gun event. In the decades that followed, the scientific community rejected the work on *Gongylonema* and condemned it as a major flaw [43, 54].

Even so, this story is meaningful in several ways. On the one hand, the *Gongylonema* studies paved the way to research into cancer causes and prevention, notwithstanding the controversy surrounding them. Secondly, it effectively illustrates the obstacles encountered in the study of cancer-causing parasites, and even nowadays it is challenging to determine whether parasites are the underlying cause of a particular malignant condition [21]. A major issue is the prolonged time frame between infection, tissue damage, and the development of malignancy. Cancer-causing parasites may no longer be present at the time of tumor diagnosis, and antibodies may fade off in cases of chronic infections, but cellular injury may eventually endure [21, 35]. Large epidemiological studies can identify an association, but there may be a number of confounding variables that could affect the evaluation of neoplastic risk. The time interval between exposure and outcome is almost unquantifiable, particularly in resource-poor settings, where both parasitic infection and cancer are unlikely to be recognized earlier. Due to limited access to healthcare, establishing registry databases for human cancers in low-income nations may be difficult and under-registration is a typical occurrence [55–59]. This is even more challenging in a veterinary medicine scenario, since global and national strategies to increase the monitoring of neoplastic pathologies are emerging only in recent years and are restricted to pets [60]. Moreover, the host spectrum variability exhibited by many parasites of veterinary importance, including *Gongylonema*, does nothing but complicate the picture further, due to species-specific pathogenetic responses to parasites [61–65].

To conclude, just as interest in the *Gongylonema* genus seemed to be waning, new data emerged. Zhou et al. [66] recently described the first human case of esophageal squamous cell cancer development linked to the gullet worm *G. pulchrum* infection. Interestingly, an association between *G. pulchrum* infection and the same neoplasia was also observed in a 17-year-old vari female (*Lemur macaca variegata*) kept in a German zoo [67]. The fact that *G. pulchrum* is phylogenetically close of to another spirurid worm, *S. lupi*, whose carcinogenic potential is widely recognized (see next section), could cast doubt on the inflexible rejection of Fibiger's work. However, given the few reports, the cancerogenic potential of *G. pulchrum* remains unknown. These data underscore the complexity of this issue and partially renew interest in Fibiger’s work, which emphasized himself the importance of multiple factors in cancer development [50].

### *Spirocerca lupi*

The nematode *Spirocerca lupi*, belonging to the Spirocercidae family, is the causal agent of spirocercosis in dogs [61, 68]. Although domestic dogs are the main hosts of *S. lupi*, various wild carnivores have also been reported to become infected, with a plethora of different clinical symptoms [61, 65, 69–74]. *S. lupi* is widespread and reports from more temperate European countries have increased in recent years, although it is common in tropical and subtropical climates [75, 76]. From a One Health perspective, variables such as climate change, urbanization, and pet travel could have altered the endemic areas of its vector, the dung beetle, and the geographic distribution of the parasite [61].

The life cycle of this parasite is quite similar to that of other spirurids, involving coprophagous beetles as intermediate hosts. Ingestion of dung beetles or other paratenic hosts carrying encapsulated L3 of *S. lupi* results in infection of the carnivore definitive hosts [61, 77]. Within two days, L3 larvae are released in the dog's stomach, penetrate the gastric mucosa, and migrate cranially through the wall of the gastric artery and the caudal thoracic aorta. In the meantime, L3 molt into fourth-stage larvae (L4). It completes its migration through the intima of the aorta to the esophagus around 100 days after infection [61, 68, 78]. The esophagus is the adult worm’s definitive localization, though abnormal migration can also take place in the thoracic cavity [79], subcutis [80], bladder [68], lungs [81], mesentery [82], or nervous system [83]. The adult parasite completes its development in the submucosa and subadventitia of the esophagus, where it induces the growth of a nodule that protrudes into the lumen of the esophagus. Female worms discharge embryonated eggs through an opening in the esophageal mucosa for up to 2 years [78, 84]. Many vertebrates can act as paratenic hosts. As a result, adult hunting and stray dogs have higher incidences of infection compared to household pets, small breed dogs, and puppies [68, 85].

A wide range of clinical symptoms may occur in cases of spirocercosis. Depending on the stage of the disease, various complications have been observed, such as vomiting, regurgitation, weight loss, dyspnea, and dysphagia, which reflect the different lesions induced by the worm in the host tissues [61, 68, 77]. These include caudal esophageal stenosis [77], thoracic vertebral spondylitis [86], hypertrophic osteopathy of the thoracic limbs [81], aneurysm formation [87], aortic iliac thromboembolism [88], hemothorax [89], and hemopericardium [90]. The most critical lesion is the growth of sarcomas in the esophagus of dogs [22, 68, 78, 91]. Malignant esophageal nodules can occur in 25% of infected dogs [92]. Osteosarcoma and fibrosarcoma are the two most frequent diagnoses [61, 78, 92], but chondrosarcoma [93] and undifferentiated sarcoma [94] can also occur. Metastases to many organs, including the tongue, lungs, kidneys, stomach, spleen, and heart have been frequently observed [23, 95].

*S. lupi* is one of the few cancer-causing parasites on which a large body of literature is available in veterinary medicine. Seibold et colleagues [91] were the first to establish a link between spirocercosis and neoplasia in 1955, reporting ten cases of esophageal sarcoma among 39 infected dogs from Auburn University (Alabama; U.S.A.), four of which were metastatic. Shortly after, Ribelin and Bailey [96] reported sixteen cases of fibrosarcoma or osteosarcoma of the esophagus linked to worm-induced lesions from the same institution. No esophageal neoplasm was found in 1806 control canine necropsies where the esophageal worm was absent. Since then, numerous additional case reports and autoptic surveys have confirmed these findings. Examples come from Israel [94], Jamaica [94], Egypt [97], Brazil [98, 99], Lousiana [100], Central America [23, 78], Kenya [72, 101–103], India [104–106]. Some of the more recent investigations were carried out in South Africa [92, 107–109], Iran [110], Bangladesh [111], Israel [95, 112, 113], and America [114–116]. Since malignant esophageal neoplasms are extremely uncommon in locations without spirocercosis [115], it was easier to prove a causal relationship [68].

The caudal esophageal region is the most affected site [78], although aorta, lung, and spinal cord sarcomas caused by *S. lupi* have also been described [51, 81, 103]. Female dogs seem to be predisposed [108]. Despite the substantial literature on the subject, it is interesting to note that no neoplastic forms have ever been identified in any of the other definitive hosts of *Spirocerca* [61].

Esophageal worm-induced nodules progress from the early inflammatory stage to the pre-neoplastic stage, and finally to the neoplastic stage. This trend has well-known histological features [107] and metabolic features [22, 61, 116]. The early inflammatory esophageal nodule first appears in the submucosa of the esophageal wall, a few centimeters cranial to the diaphragm [23]. Smooth nodules contain several adult male and female parasites, as well as a significant number of neutrophils, fibrocytes, a lesser number of lymphocytes and abundant collagen deposition [107]. These nodules have been sometimes wrongly referred to as granulomas, but they lack structured macrophages and mostly contain neutrophils [61, 78, 107]. In contrast to pre-neoplastic nodules, which include immature proliferating fibroblasts and a smaller amount of collagen, the normal connective tissue is mainly made up of fibrocytes and significant amounts of collagen. Moreover, pre-neoplastic nodules have fewer parasites, a higher mitotic index, and more multinucleated cells. Adult worms, which are located in the surrounding connective tissue near small purulent areas, are rarely detected in the lesions during the neoplastic stage [117]. The malignant nodule can grow up to 11 cm in length, and displays necrosis, after losing its smooth look [107, 118].

In conclusion, *S. lupi* is the sole nematode that has been confirmed to cause malignant processes in dogs [22]. This parasite has been suggested as a model to research how nematodes operate as carcinogenic agents. However, the literature often ignores this parasite due to its scanty zoonotic potential [21, 29, 30], and issues remain in the in vivo experimental induction of cancer by *S. lupi* in laboratory animals (since they act as paratenic hosts), and in canids (for ethical reasons) [119]. Attempts have recently been made to maintain adult *S. lupi* worms alive in ex vivo murine fibroblasts cultures, albeit for a short period of time [120]. Still, the expanding area of endemicity of this parasite and the scarcity of cancer-causing nematodes in human medicine makes it particularly intriguing, and further studies are undoubtedly warranted.

### *Heterakis gallinarum* and *Heterakis isolonche*

Members of the genus *Heterakis* are common Ascaridida parasites that reside in the ceca of numerous poultry bird species. There are several *Heterakis* species that fall under this taxon, but since molecular diagnostics have not been frequently employed to analyze populations of *Heterakis* in the field, it is challenging to precisely identify these species or establish their phylogenetic relationships [121]. Moreover, there is considerable overlap in the host species that are receptive to each *Heterakis* spp. strain, and concurrent coinfections have been documented [122–124].

The direct life cycle of this parasite contributes to its high prevalence in intensive flocks. Adults reproduce in host ceca. Unembryonated *Heterakis* eggs are expelled by gravid females in the lumen of the cecum, and they are shed in feces. Environmental factors such as temperature, humidity, and aerobic conditions outside the host influence egg development into infectious L2 larvae over the course of around two weeks [125]. Larvae can be ingested directly by a specific host or by a paratenic host like an earthworm [122, 123].

The traditional main health concern associated with *Heterakis* parasites is that one species, *H. gallinarum*, is crucial in the spread of the protozoan parasite *Histomonas meleagridis*, which is the primary cause of a severe condition known as "blackhead" in Galliformes [126, 127]. *H. gallinarum* does not migrate inside the host’s tissues, and only modest lesions with negligible impact on bird performance are usually recorded [123, 124]. However, cecal nodules due to worm irritation can be observed, especially in cases of subsequent or heavy infections [123, 124, 128–130]. Nodular or verrucous typhlitis associated with *Heterakis* occurs worldwide in various flock bird species, mainly pheasants, and is characterized by the development of inflammatory, granulomatous, or even neoplastic nodules in the cecal wall, primarily in the submucosa. This condition is mainly caused by the nematode *H. isolonche* [124, 131]. The association between *Heterakis* infection and mesenchymal malignant transformation was first observed in Italy at the end of the nineteenth century [132]. The Italian veterinarian pathologist Galli-Valerio identified a fibrosarcoma in the cecum of a *H. gallinarum*-infected green pheasant (*Phasianus versicolor*), making this parasite one of the first ones hypothesized to be associated with cancer. Subsequently, other reports described mesenchymal tumors caused by *H. isolonche* in the common pheasant (*Phasianus colchicus*) [133] and the golden pheasant (*Crysolophus pictus*) [134]. Next, in 1972 Helmboldt and Wyand [135] described the presence of leiomyomas in three golden pheasants caused by *Heterakis* species. Each nodule included *Heterakis* worm fragments that could be recognized by their morphology, coupled with clusters of reactive lymphocytes. The authors described clear progression from inflammatory to neoplastic tumors. *Heterakis gallinarum* was grossly found free in the cecal lumen of two of these pheasants.

Balaguer et al. [136] and Menezes et al. [124] conducted two more recent studies. In the first one, four cases of nodular typhlitis caused by *H. isolonche* were found in a multispecies flock of pheasants (*Crossoptilon aritum, C. mantchuricum*, and *Syrmaticus soemmerringii*) from a Spanish Zoo*.* The cecal walls of all four bird species revealed extensive lobular thickening because of multiple, whitish, firm nodules that ranged in diameter from 1 to 3 mm. Several nodules and the cecal lumen contained *H. isolonche* nematodes. The neoplastic cell morphology and immunostaining pattern were consistent with benign tumors of the smooth muscle (leiomyoma) [136]. In the other study paper, an epidemiological and pathological survey was carried out on 50 ring-necked pheasants (*Phasianus colchicus*) from enclosures in Rio de Janeiro, Brazil [124]. A high prevalence (90%) of *H. gallinarum* infection, and absence of *H. isolonche*, was found. Chronic diffuse typhlitis, granulomas, and necrotic foci surrounded by myxoid matrix, hemosiderin deposits, lymphocytic infiltrates, multinuclear giant cells, and histiocytes in the submucosa of the cecal wall were found linked to immature *H. gallinarum* worms. Atypical fusiform mesenchymal cells with elongated nuclei clustered in chaotic whorls and dense bundles, low mitotic figures and infiltrative aspects, and absence of metastasis lesions met the diagnostic criteria of a leiomyoma in eight animals [124]. Surprisingly, unlike phlogistic lesions, neoplastic change was unrelated to parasite burden [123, 124]. Persistent *H. gallinarum* reinfections might lead to an intratissue parasitic phase [128, 130], where granulomatous nodules could further progress to neoplasia [124]. Sequential infection by different nematode strains transmitted by other gallinaceous species might also lead to the same effect. In addition, previous research had revealed that certain *Heterakis* strains are not adapted to pheasants and may trigger higher pathogenicity [63].

In light of these results, the host-parasite relationship between several species of poultry and *Heterakis* strains may serve as a common and widespread animal model to shed light on the inflammatory-to-neoplastic pathway [22, 124].

### *Ophidascaris* spp.

Besides *Heterakis*, another member of the Ascarididae family has been linked to a malignant condition. Four nematodes were discovered inside necrotic areas of gastric adenocarcinoma in a 7-year-old male diamond python (*Morelia spilota spilota*) in a recent paper by Baron et colleagues [137]. *Ophidascaris* spp. was identified morphologically and by molecular investigations. Many snake species are commonly infected with nematodes of this genus [138]. The adult parasites develop nodular masses in the esophagus and stomach of snakes, embedding their anterior bodies into the digestive mucosa. The L3 larvae are normally located in the liver of the intermediate host, such as small rodents or marsupials [139]. The mechanisms that predispose snakes to develop neoplasia are still poorly understood. Arenaviral and retroviral infections, among other viruses, have been suggested to cause a number of snake tumors [140, 141]. The case report by Baron is the lone and first paper concerning this parasite etiology. Snake adenocarcinomas are rather common tumors [142] and so is *Ophidascaris* infection [138, 143]. Hence, parasite infection might have been an independent event occurring by accident in a snake with adenocarcinoma. Yet, the bordering of the non-encapsulated, infiltrative epithelial neoplasm with a significant desmoplastic response and the numerous granulomas coupled to the nematodes embedded within the gastrointestinal wall suggested that the inflammatory response to ascaridiasis had an oncogenic role in the development of the neoplasm described [137].

### *Ollullanus trichuspis*

A second report concerning nematode infection in gastric adenocarcinoma growth was published by Dennis et colleagues [144]. The concomitant development of gastric adenocarcinoma in two neutered male Persian cats was observed at Colorado State University. Interestingly, the two cats were of the same lineage and had been raised together in an indoor setting. A transmural mass with a core area of necrosis in the fundus region, with polypoid consolidating projections on its surface was surgically biopsied. At histology, tubuloacinar structures infiltrated the deep *lamina propria,* submucosa, and *muscularis mucosae* with lymphovascular invasion. Metastases to the liver, kidney, and lungs were also reported. At the periphery, fibrosis and proliferative gastritis with intralesional *Ollullanus tricuspis* worms were highlighted [144]. These worms are small parasitic Strongylida from the Trichostrongylidae family that reside in the stomach of cats and other felids, with a worldwide distribution [145]. Infections in dogs have also been reported [146]. *Ollullanus tricuspis* has a direct life cycle, and its transmission takes place by ingestion of infected cat vomitus by other hosts. While eggs and larvae are not generally shed in the feces, any stage of the parasite life cycle can be transmitted by this route [145, 146]. *Ollullanus tricuspis* is thought to have a typically low pathogenic potential, causing mucosal erosions, increased mucus production, and hyperplasia of the lymphoid follicles [144]. However, life-threatening sequelae have been reported, including severe chronic gastritis [147, 148]. No additional cases of simultaneous gastric adenocarcinoma developing in two cats of the same age and belonging to the same household are reported in the literature. In fact, only occasional reports exist for this type of tumor, which has a very low incidence in the feline species [149, 150]. Thus, given the uncommon nature of this scenario, parasite-driven chronic gastritis may have contributed to the onset of stomach adenocarcinoma in these two related cats, in conjunction with other environmental and genetic variables.

### *Nochtia nochti*

Among the members of the Trichostrongylidae family, *Nochtia nochti* is a small, bright red worm that has been observed in the prepyloric region of the stomach of Old World monkeys, especially macaques (*Macaca* spp.) [151]. Its life cycle is direct, with L3 larvae intake by fecal–oral transmission. The parasites burrow into the gastric mucosa at the fundus-pylorus junction, mature into adults, and lay eggs shed with feces, which become infectious L3 larvae after a week [152].

*Nochtia nochti* infection has been causally linked to epithelial stomach cancers in six crab-eating macaques (*Macaca fascicularis*) in 1939 by Bonne and Sandground [153]. The presence of worms was observed in each, and no *N. nochti* was found in the absence of a tumor. A causal role for the parasite was experimentally confirmed: when adult *Nochtia* worms were introduced into the stomach of two healthy monkeys, tumors occurred within three months [153]. The lesions emerged from the gastric mucosa at the border between the prepyloric and fundal sections as hyperemic, cauliflower-like masses, initially appearing as benign papilloma, but closer examination revealed a more malignant behavior. The *muscularis mucosa,* the submucosa, and the muscular layer were frequently invaded by proliferating epithelial cells. Lymphovascular invasion at the periphery of the lesion was reported too [153]. Further studies confirmed the association of this parasitic agent to gastric benign papillomas in a wider number of stump-tail macaques (*Macaca speciosa*) [154], crab-eating macaques [155–157], and rhesus macaques (*Macaca mulatta*) [157, 158], although their true nature is a matter of debate. In fact, unlike what was reported by Bonne and Sandground [153], no evidence of malignancy was reported in these cases. Consequently, some authors believe that the growth behavior of the epithelial cells is more likely to be reactive hyper-regeneration rather than neoplastic proliferation [154, 156, 158], similar to other proliferative gastritis forms induced by trichostrongylids [159, 160], oxyurids [161], capillarids [162], and *Physaloptera spp*. [152] in other primate and non-primate species.

### *Trichinella* spp.

Trichinellosis is a parasitic disease caused by nematodes belonging to the Trichinellidae family, genus *Trichinella*. They can infect humans as well as more than 150 other ectothermic and homeothermic animals, making it the most common food-borne helminth zoonosis [163]. In addition to the significant impact this parasite had [164] and continues to have on public health [165, 166], it also represents a very intriguing and paradoxical research area for cancerogenesis and is being studied on two apparently antithetical fronts. On the one hand, evidence of *Trichinella spiralis* anticancer activity has been provided by numerous researchers. Its potential anticancer pathways were recently reviewed in a comprehensive manner by various authors [167, 168] and will be mentioned in the last part of this paper. On the other hand, since the underlying mechanisms of host-parasite balance are still unclear, several additional studies have suggested that *T. spiralis* may be a cause or a contributing factor to tumor development in humans and animals [167, 169, 170].

Trichinella has an unusual but very basic life cycle. Once meat carrying tissue cysts has been consumed, the intestinal phase starts. Gastric juices and acid in the gut release muscle larvae, which reach the small intestine after a few hours and invade the enteric mucosa. There, they go through four molts to reach the adult stage and start mating as soon as two days after infection. Larvae move to the striated muscle via the circulatory system. Muscle fibers are then reprogrammed by some *Trichinella* species to form a capsule (nurse cells), where the infectious larvae mature [163].

The relationship between *Trichinella* spp. with human cancer was first addressed decades ago. Lewy et al. originally described a case of laryngeal cancer associated with *Trichinella* larvae in 1964 [171]. Further research revealed that this type of relationship occurs frequently in head and neck malignancies, such as oral cancer, tongue carcinoma, and laryngeal cancer, with squamous cell carcinoma being the most common neoplasia identified [172–175]. *Trichinella* larvae prefer to reside in the jaws, tongue, throat, and eyes, therefore chronic inflammation of these muscles was first thought to be the main contributing factor. Nonetheless, *Trichinella* has occasionally been linked to other tumor types [176].

Cases of *Trichinella*-associated cancer have also been recorded in veterinary medicine, albeit on a smaller scale. Moisan et al. [170] reported trichinosis and oral squamous cell cancer in a 10-year-old domestic shorthair cat. Like human oral malignancies, several *Trichinella* larvae were observed in laryngeal muscles and within neoplastic tissue. A few years later, another case where trichinosis was presumed to cause a non-healing ulcerative lesion involving the eyelid and conjunctiva of an 8 years-old domestic shorthair cat was described by Saari et al. [177]. Phlogistic lesions were prevalent, but mesenchymal neoplastic cellular features identified by histology suggested low-grade fibrosarcoma as a differential diagnosis. Previously, a *Trichinella*-associated melanoma arising from the eyelid in a dog was observed [169]. The limited number of case reports makes it impossible to rule out an independent relationship between parasitic localization and neoplastic process, but the similarities between feline, canine, and human pictures continue to raise questions about the potential of *Trichinella* for cancerogenesis.

### *Clonorchis sinensis*

Liver flukes are a type of widespread zoonotic platyhelminths that can cause liver and bile duct disorders. The Opistorchiidae family, which includes *Clonorchis sinensis*, *Opistorchis viverrini*, and *O. felineus*, can have adverse effects on both human and animal health, with nearly 45 million people infected by these fish-borne liver parasites [32, 178, 179]. *Clonorchis sinensis* is endemic in Asian countries such as China, the Republic of Korea, northern Vietnam, and far-eastern Russia. More than 15 million human infections by this species are currently estimated [180, 181].

The parasite has a three-host life cycle, with humans becoming infected by consuming raw fish containing cysts (metacercariae) in the muscles and connective tissues [182]. After being excysted in the human duodenum, the metacercariae travel along the bile duct epithelial lining and mature into adult worms, primarily within the intrahepatic bile channels within a month. The adult fluke attaches to the bile ducts using a pair of powerful suckers and produces eggs that are passed into feces. *Bithynia* spp. snails, the first intermediate hosts [183], consume the eggs, which then develop and exit the snails as cercariae. The cercariae enter the second intermediate host, a cyprinoid fish. Piscivorous animals, particularly cats and dogs, act as reservoir hosts for *C. sinensis* and play a role in the epidemiology of the parasite since they are widespread animals and can sustain the parasite lifecycle without human involvement [184]. Our understanding of this parasite has significantly increased since James McConnell discovered *C. sinensis* in the bile ducts of a young Chinese man in 1875 [185]. Inflammation, epithelial and adenomatous hyperplasia, mucinous metaplasia, cholangiofibrosis, and granuloma development are the primary histological features of liver fluke infection in humans [186].

In 1994, *C. sinensis* was classified as a probable carcinogen to humans (Group 2A). However, thanks to more recent and solid evidence [180, 187] it is currently classified as a highly carcinogenic agent to humans (Group 1) [5]. Cholangiocarcinoma is the most dangerous complication of clonorchiasis: adenocarcinomas make up 70% of *C. sinensis*-induced cancer, while bile duct anaplastic and squamous tumors comprise the rest [180, 181, 188]. Recent studies indicate that patients with hepatocellular carcinoma and *C. sinensis* infection have a worse prognosis, regardless of HBV co-infection [189]. Experimental animal models have helped to understand carcinogenic mechanisms [190], while there is limited information in the literature for domestic animals. Three papers written by Hou and colleagues were the first to point out the similarities between human, canine, and feline *C. sinensis*-induced cancers [191–193].

In a study conducted by Hong Kong University, three cases of feline cholangiocarcinoma were examined, two of which were spontaneous and one was experimentally induced. The study revealed thick-walled second-order bile ducts filled with C. sinensis in all three cases [191]. Malignant cells, clearly originating from hyperplastic adenomatous tissue, were observed along with severe fibrosis. Local progression to the diaphragm and metastases to hepatic lymph nodes were also noted [192]. Similar histopathological lesions were found in an 8-year-old female Chow dog by the same group, which supports the parasitic etiology of *C. sinensis*-induced cancer in dogs, as well as in humans and cats [193]. As the development time for this type of cancer is strongly correlated with the lifespan of the host, there appears to be an age cut-off for cats and dogs as well, since all cancer cases developed in middle-aged individuals [192, 193]. This may help to explain the scarcity of such reports, along with the limited availability of veterinary diagnostic services in highly endemic areas.

### *Opistorchis* spp.

Another member of the Opistorchidae family is the carcinogenic liver fluke *O. viverrini*. This trematode is widespread in Southeast Asia, primarily in the Mekong River region, where it affects over 8–10 million people [56]. Like *C. sinensis,* its life cycle involves cyprinid fish as the second intermediate hosts and aquatic snails of the genus *Bithynia* as the first intermediate hosts. While humans are thought to be the primary definitive hosts [194], cats and dogs serve as reservoir hosts for *O. viverrini* and help to spread it [195].

Opisthorchiasis is undeniably associated with the development of cholangiocarcinoma, as per numerous descriptive studies and extensive epidemiological surveys. Indeed, it is classified as a Group 1 carcinogen by IARC [5, 32]. Due to the *O. viverrini* adult worm’s ability to survive in the human liver for over 10 years, many chronic infections lead to the growth of malignant biliary duct tumors [56].

Even though any fish-eating mammal might be infected, *O. viverrini* infections in animals are rare [9]. Cats have a higher prevalence of *O. viverrini* infection (35.51%) than dogs (0.37%) in Thailand [196], but the spread of this parasitism is often underestimated [195, 197]. In a case report from Thailand, a 12-year-old female domestic cat was found to have multifocal, cystic, whitish liver tumors with trematodes inside the biliary ducts [198]. Histology revealed a biliary cystadenoma with irregular, well-differentiated epithelial cells, multifocal trematodes located in several cysts, and infiltration of eosinophils and mononuclear cells. Molecular analysis confirmed the involvement of *O. viverrini* [198]. This is the only case report documenting the spontaneous formation of biliary cystadenoma in an *O. viverrini*-infected cat, despite the widespread distribution of the parasite. Like *Clonorchis* sp*.*, various biological factors such as the life expectancy of piscivorous hosts, and coevolution of different *O. viverrini* strains with human or feline definitive hosts may be involved in this paradoxical picture [195]. Socio-sanitary elements, such as public health surveillance and access to standard diagnostics could be at play as well [195].

*Opisthorchis felineus*, the "European liver fluke", is a close relative of *O. viverrini* also suspected of having cancer-causing properties. The first description of *O. felineus* was provided in 1884 by Italian scientist Sebastiano Rivolta, who called the parasites "*Distoma felineum*" after detecting them in cats and dogs in Pisa (Italy) [199]. The geographic range of *O. felineus* spans from Western Siberia to Mediterranean Europe [9, 200, 201]. Human infection is most common in Russia, where up to 40 thousand infections are detected each year [9].

*Opistorchis felineus* circulates across Europe thanks to piscivorous domestic and wild animals, with scant human involvement until hazardous eating behaviors and eating habits occur [199, 201, 202]. According to epidemiological research, this fluke infection is correlated to severe hepatobiliary illness, as well as being a risk factor for cholangiocarcinoma [9, 200, 203]. Experimentally-induced cancer in rodent models shows that *O. felineus* carcinogenic properties are similar to those of *O. viverrini* and *C. sinensis* [62, 204–207]. However, no natural-occurring cases of biliary neoplasia in other non-human definitive hosts have been reported to date [13]. Thus, the differences between *O. felineus* and its well-known cancer-causing relatives and the mechanisms by which this parasite might cause neoplastic diseases are still widely unknown. Because there is still insufficient epidemiological evidence to support a causal relationship between this parasitosis and biliary tract cancer, IARC classified *Opisthorchis felineus* as belonging to Category 3, which includes potential cancer-causing substances for which there is insufficient evidence of their ability to cause cancer in both humans and animals [5, 9, 32, 203].

### *Fasciola* spp.

Fasciolosis is a parasitic zoonosis that is often neglected in developing countries and is caused by the infection of liver flukes belonging to the family Fasciolidae, specifically *Fasciola hepatica* and *F. gigantica* [208]. The life cycle of these flukes is indirect, with the intermediate freshwater snail host being infected by miracidia that hatch from eggs shed in feces by the definitive host. After reproducing asexually, the cercariae emerge from the snail and develop into infectious metacercarial cysts attached to aquatic plants. A wide range of vertebrate animals can act as definitive hosts by ingesting the metacercariae, leading to widespread transmission and circulation [209]. Domestic livestock and wild species are the reservoirs for these parasites across Asia, Africa, Europe, and the Americas [210, 211]. Since reports of *F. hepatica* infection in humans are more frequent than those of *F. gigantica*, it is believed that *F. hepatica* has a higher potential for zoonotic spread. Yet, due to limited access to healthcare in endemic areas, cases of human infection may be overlooked [208, 212].

The parasite burden is related to hepatic lesions in various host species. Mechanical injury and inflammation are linked to the migration of juvenile flukes through the intestinal wall, abdominal cavity, and liver parenchyma, as well as the feeding behavior of adult flukes in the bile ducts [213]. Although *Fasciola* spp. are closely related to the previously described liver trematodes, there is yet no conclusive evidence linking these parasites to human cancer [207]. Nonetheless, this fluke has occasionally been linked to human and animal disease complications, mostly as a cause of liver fibrosis and cirrhosis, comparable to what has been described in other closely related veterinary-relevant agents, such the giant liver fluke (*Fascioloides magna*) [210, 211, 214, 215]. The role of *Fasciola* in carcinogenesis has been hypothesized in two separate neoplastic entities in veterinary medicine. In an intriguing case–control study, Bahrami et al. [216] investigated the histological and clinicopathological modifications in 49 *F. gigantica*-infected and 20 healthy cattle from the southwest of Iran. In 10 liver samples from infected animals, multifocal homogenous populations of tightly packed spindle cells with blunt-ended nuclei oriented in interlacing fascicles were consistent with leiomyoma [216]. Previously, only one case report presenting a case of leiomyoma in the end-stage liver of a cow with fascioliasis had been published in the literature [217]. Tortuous fibrotic areas, cirrhosis, and chronic catarrhal cholangitis were the most common lesions caused by *Fasciola* [216, 217]. In addition, a recent paper by Shahvazi et al. [218] from the same area reported leiomyomas in 44 out of 50 (90.0%) infected cattle, confirming previous results.

On the other hand, in southern Bohemia, a *F. hepatica* endemic area, Vitovek and colleagues found hepatocellular carcinomas in 11 livers from cattle in a slaughterhouse [219]. The presence of both cancer and *Fasciola*-induced biliary cirrhosis in every case raises the possibility of a causative relationship. The higher incidence of this neoplasm in the sampled population and the common left lobe localization (in which *Fasciola* is preferentially localized) support the hypothesis.

In humans, hepatocellular carcinoma is a well-established sequela of liver disorders, such as hepatitis or cirrhosis. However, a similar link is not yet clearly recognized in veterinary medicine [219, 220]. No evidence of cancerogenesis induced by *F. hepatica* and *gigantica* was reported in humans, except for in vitro studies [210, 212] and a recent case report of *F. hepatica* infestation in an Indian female with inoperable gallbladder carcinoma [221]. Thus, a deeper look into this topic in animals could help in the comprehension of this phenomenon.

### *Platynosomum illiciens*

*Platynosomum* is a genus in the Dicrocoeliidae family of biliary trematodes that parasitize birds and mammals distributed worldwide. The parasite found in South American cats was previously considered a separate species (*Platynosomum fastosum*) for many years, but is now considered a synonym of *P. illiciens* after a recent taxonomic revision [222, 223]. This parasite also infects non-felid hosts, which complicates the epidemiologic scenario [222]. The life cycle of this parasite requires three intermediate hosts: a mollusk where embryonated eggs develop into sporocysts containing cercariae; a terrestrial isopod, where the cercariae are released and develop into metacercariae (the infective form); and a small vertebrate paratenic host, such as a lizard or toad. In the definitive hosts, which are generally asymptomatic, adult trematodes localize in the choledochus and gall bladder [224]. While most papers reporting this parasitism in different parts of the world did not describe cancerogenesis [222, 225, 226], seven cases of cholangiocarcinomas in adult cats parasitized by *P. illiciens* were reported in Brazil [227, 228]. The liver tumors were composed of atypical neoplastic cells arranged in acini, and metastases to various organs were noted, with visible pre-neoplastic alterations resembling proliferative or chronic inflammatory lesions seen in other trematode-induced malignancies [21]. Furthermore, a recent case of spontaneous hepatocellular carcinoma in a free-living adult male marmoset (*Callithrix* sp.) was recently observed in combination with *Platynosomum* sp*.* infection in Brazil [229]. Although a direct cause-effect link may be incidental, the authors could not rule out a potential primary or contributing oncogenic role of the parasite.

### *Schistosoma mansoni*

Schistosoma, a genus of blood flukes, is responsible for infecting 236 million people worldwide, primarily in low-income countries [230]. Among the various species, only *S. haematobium* is classified as a Group 1 carcinogen by IARC. In contrast, *S. mansoni* infection is listed as Group 3 [5, 8]. The gonochoric adult flukes inhabit the mesenteric veins close to the intestine, and infection with intestinal schistosomiasis occurs when cercariae, which have developed in freshwater snails (intermediate host), penetrate human skin. Despite the regular occurrence of *S. mansoni* in wild primates living in endemic regions, it is not considered a zoonosis because it primarily affects humans [231]. A single report has associated chronic *S. mansoni* infection with spontaneous cancer in a 12-year-old chimpanzee (*Pan troglodytes*) born in Sierra Leone [232]. Histological analysis of the liver tumor, which presented as a single hard nodule, revealed a well-differentiated hepatocellular carcinoma with a trabecular pattern. Serological testing ruled out infections with HBV and HCV [232]. Other experimental and clinical reports suggest that this parasite may increase the risk of various human malignancies, including hepatocellular carcinoma, colorectal cancer, bladder carcinoma, prostate cancer, and follicular lymphoma [8, 32, 215].

### *Taenia* (*Hydatigera*)* taeniformis*

Tapeworms are flatworms belonging to the phylum Platyhelmintes that can inhabit the digestive tract of several species. Infections with tapeworms have significant veterinary and medical repercussions [233]. Members of the family Taeniidae (*Echinococcus*, *Taenia*, and *Versteria* spp.) require two mammalian hosts in a predator–prey or scavenging relationship to complete their life cycles [234, 235].

Among these, *T. taeniaeformis* is one of the most widely studied and distributed tapeworms in veterinary medicine, primarily infecting cats (definitive hosts) and rodents/lagomorphs (intermediate hosts) [236, 237]. Although isolated cases of human infection have been reported, the zoonotic potential is yet unknown [238, 239]. Cats excrete *T. taeniaeformis* eggs into the environment, which are then consumed by the intermediate hosts. The eggs then progress to the metacestode stage (*Strobilocercus fasciolaris* or *Cysticercus fasciolaris*), leading to the development of liver cysts [235].

Although infections in rats are frequently subclinical, the cancerogenic potential of *T. taeniaeformis* is widely recognized. This parasite has been linked to the development of primary hepatic fibrosarcoma in rodents [52, 240–249]. Additionally, benign hepatic fibromas have also been reported [241, 250].

In the early 1900s, it was first hypothesized that liver tumors in rats could develop in conjunction with the implantation of larvae [52, 241, 242]. Within a few years, three independent physicians described the same pathological findings in laboratory rats (*Rattus norvegicus*). Borrel described two tumors in the kidney and in the liver of two rats that died in two different labs. In both cases, the tumors were attached to a cystic sac containing hyaline fluid and metacestodes identified as *S. fasciolaris* [52]. Concurrently, during the systematic examination of thousands of wild rats killed in a campaign for the eradication of plague at the Federal Plague Laboratory in San Francisco, McCoy found 11 sarcomas and one fibroma associated with *S. fasciolaris*, all affecting the liver [241]. A few years later, Woolley and Wherry confirmed such findings by reporting 3 hepatic sarcomas coupled with *S. fasciolaris* infection in wild rats [242]. The three papers often described metastases to lymph nodes, mesentery, and local invasion.

In numerous experiments, Bullock and colleagues exhaustively confirmed malignant transformation of the capsular connective tissue surrounding the encysted larvae in laboratory rats [251–257]. Further in vitro and in vivo experimental infections helped in the comprehension of the transforming mechanism [239, 258–260]. Spontaneous *T. taeniformis*-induced neoplastic cases have also been reported in wild [244], laboratory [240, 245, 248–250], and pet rats [247]. In such investigations, large chronic-stage hepatic cysts, inflammation featuring granulation tissue, Kupffer cell infiltration, and scattered lymphocytes were described. The typical hallmarks of primary fibrosarcoma were pleomorphic fibroblasts in disorganized and centrifugal bundles invading the adjacent liver from the capsule, which were described in up to 73% of infected animals [240, 248, 249].

Despite the involvement of numerous intermediate species in the life cycle [261–264], the vast majority of cases of hepatic fibrosarcoma have been identified in rats, with only a few cases reported in mice (*Mus musculus*) [246] and muskrats (*Ondatra zibethicus*) [243]. These findings suggest that the predisposition to oncogenesis is partly determined by the host species. Furthermore, an outstanding question is why so few neoplasms in wild rather than laboratory rats are reported, despite thorough examination [265–271]. Most tumors are thought to develop between 11 and 17 months after infection [245, 247, 252, 254]. However, younger rats (5–8 months old) were shown to exhibit hepatic fibrosarcoma by Mahesh Kumar et al. [240], and Al-Salihi et al. [248]. Although this issue is still unclear and lab rats’ lifespans might vary significantly, it is conceivable that the prolonged post-infection carcinogenic timeframe and the shorter average life expectancy of free-living rats (which is often shorter than one year) may play a role [162, 272].

### *Linguatula serrata*

The subphylum Crustacea comprises zoonotic parasitic arthropods called pentastomes, also known as tongue worms, which exhibit wormlike characteristics. One of these parasites, *Linguatula serrata*, has a global distribution and primarily infects carnivores as definitive hosts [273]. The adult parasites reside in the nasal cavity, where they lay their eggs that are excreted in feces and nasal mucus. Several intermediate hosts, such as fish, rodents, and ungulates, can harbor infectious visceral larvae after ingesting the eggs. Humans may inadvertently serve as intermediate or final hosts following the consumption of uncooked meat, contaminated water, or raw vegetables [273].

In Italy, Bordicchia et al. reported a case of a 6-year-old mixed-breed dog with nasal basosquamous cancer and concurrent linguatulosis [274]. During autopsy and histological examination *L. serrata* nymphs were found in the neoplastic tissue, accompanied by a granulomatous reaction. Given the severe phlogosis observed, a causal involvement of the parasite was hypothesized. Scant reports on pentastomes and cancer have been published in both veterinary and human medicine. Pentastomiasis was diagnosed in a 20-year-old female oriental small-clawed otter (*Aonyx cinereus*) with thyroid gland carcinoma [275], in a boy with acute leukaemia [276], and in a man with metastatic thyroid cancer [277]. Except for the dog described by Bordicchia et colleagues the most probable scenario in these instances appears to be secondary infection resulting from neoplastic debilitation.

### *Theileria annulata* and *Cryptosporidium* spp*.*

A fascinating association between parasitic protozoan infection and cancer was highlighted in the case of the bovine piroplasm *Theileria*. *Theileria annulata* and *Theileria parva* are tick-borne haemoprotozoan Apicomplexan parasites. Tropical theileriosis, caused by *T. annulata* and involving macrophages, dendritic cells, and B cells, is a fatal leuko-proliferative disease of cattle [278, 279]. *Theileria parva*, on the other hand, preferentially infects T cells (and less frequently B cells), resulting in East Coast fever [278–280]. These parasites have a high mortality rate if left untreated [281], and *T. parva* alone kills more than one million cattle annually in sub-Saharan Africa [280]. However, no *Theileria* species have been identified as causing zoonotic diseases [282].

These protozoa are obligate intracellular pathogens, where parasites and host cells are inextricably linked. As a result, these species have developed mechanisms of evolutionary advantage causing hyperproliferation, immortalization, and dissemination of the parasitized target cells [279, 283, 284]. Such a scenario fits with all the features defining a neoplastic phenomenon, except that it is reversible [27]. Indeed, etiological treatment with theilericidal drugs almost entirely reverses the process [278]. Sporozoites are transmitted to domestic or wild ruminant hosts by ixodid ticks. Macrophages or lymphocytes are invaded by *Theileria* sporozoites, which develop in a multinucleated schizont attached to the microtubule organizing center (MTOC) directly in the host cell cytoplasm [279, 285]. The cytoplasmic schizont can turn its host leukocyte into a continuously replicating tumor-like cell, promoting parasitic spread. Due to its close association with the MTOC, the intracellular schizont is partitioned into both daughter leukocytes during each host cell division [285]. During spread, a number of schizonts produce merozoites that penetrate red blood cells, circulate in the bloodstream as piroplasms, and are ingested by feeding ticks, where they produce new infectious sporozoites [27, 278, 279].

Typically, fever, anorexia, lymphoadenomegaly, respiratory distress, acute anemia, and jaundice are the major clinical signs, whose severity varies amongst different cattle breeds [280, 286]. Several organs may be infiltrated by schizont-transformed leukocytes resulting in thrombosis, parenchymal necrosis, hemorrhage, and inflammation [280, 287].

Combined infection with bovine leukaemia virus (BLV) and *T. annulata* was documented in a 6-month-old calf with a diagnosis of lymphoma [288]. Pleomorphic lymphoblasts were seen in swollen lymph nodes, heart, and omentum. The authors claim that whereas BLV infection primarily affects adult cattle, its occurrence in a calf could be attributed to concurrent *Theileria* infection, which may have hastened the neoplastic transformation brought on by the retrovirus [288].

There has been a resurgence of interest in *Theileria* as a research model to shed light into the molecular pathway involved in neoplastic transformation. *Theileria*-infected cells exhibit uncontrolled proliferation and enhanced metastatic potential in rodents [289–291], providing a wonderful example of how intracellular parasites interact with their host cells to convert the cell phenotype [279, 284].

Except for *Theileria*-driven proliferation, there is little information on other protozoa of veterinary relevance and cancer. Nevertheless, the zoonotic intracellular protozoa *Cryptosporidium* spp*.* have recently attracted interest [11]. The entire life cycle of *Cryptosporidium* spp. takes place in the gastrointestinal tract of a wide variety of animal hosts, and infection occurs mainly by ingestion of sporulated oocysts in fecally-contaminated water and food [11]. Cryptosporidiosis, being associated with the development of colorectal cancer in humans [35, 38, 292], may contribute to the development of malignant tumors. Indeed, an association between *Cryptosporidium* spp. has already been described in experimentally infected immunodeficient mice [293, 294].

In spontaneous settings, two papers [295, 296] described a link between aural-pharyngeal polyps and *Cryptosporidium* spp. in iguanas. Evidence of *Cryptosporidium* spp. coupled with proventricular metaplasia in snowy owls (*Bubo scandiacus*) was found, too [297]. The presence of neoplastic lesions was not described in any of these investigations.

*Theileria* and *Cryptosporidium* are examples in which further research from a veterinary perspective would be welcome to improve our understanding of protozoan-induced cancer.

## Carcinogenic mechanisms

**Fig. 1:**
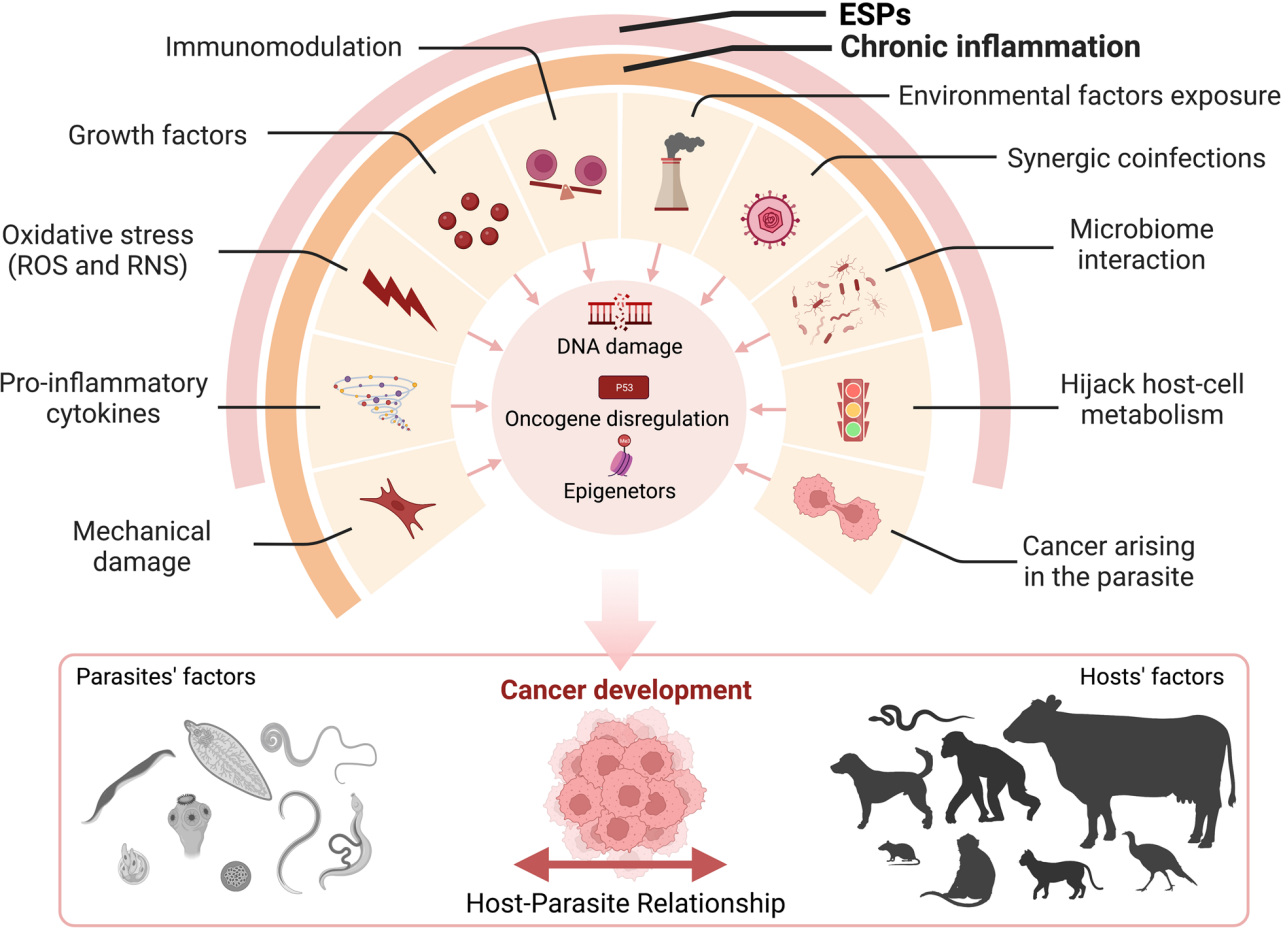
Schematic summary of cancerogenic mechanisms involved in human and animal parasitic infections. ESPs, excretory and secretory products; ROS, reactive oxygen species; RNS, reactive nitrogen species. Created with BioRender.com

Cancer development is a multistep process caused by aberrant gene expression via genetic and epigenetic mechanisms, which results in neoplastic cell initiation, promotion, and finally progression [298]. In recent years, there has been significant improvement in the knowledge of the molecular mechanisms that guide the host-parasite interaction [299], also thanks to numerous in vitro and in vivo studies [11, 22]. This has led to a significant number of hypotheses relating to how this relationship can drive neoplastic development, and consequently to potential therapeutic targets. Several pathogenic mechanisms, even bizarre ones, are proposed to explain the development of parasite-related tumors. Figure 1 displays a schematic summary of these mechanisms. Many cancerogenic pathways, particularly regarding human carcinogens, have been carefully reviewed elsewhere [6–8, 29, 30, 35, 300–303]. The next sections will provide a brief overview of such mechanisms in veterinary-related parasites.

### Chronic inflammation

Around 150 years ago, Rudolph Virchow first proposed a link between inflammation and cancer [304]. Since then, several authors have investigated this relationship, and increasing evidence suggests that inflammatory processes are involved in all the phases of carcinogenesis [305–309]. Over nearly the entire twentieth century, chronic inflammation has been the most widely accepted parasite-associated cancerogenic mechanism. In all the veterinary cancer-causing parasites reported in Table 1, an inflammatory role in cancerogenesis was postulated [67, 107, 124, 137, 144, 153, 167, 215, 247, 274, 279]. A clear continuum between inflammatory and neoplastic lesions, especially for *S. lupi*, *Heterakis* spp., *T. taeniformis*, and liver fluke infections, is immediately evident [107, 124, 247].

Inflammation promotes cancer in a variety of ways: physical damage, oxidative stress, and the release of mediators like cytokines, prostaglandins, and growth factors can cause DNA damage in tumor suppressor genes, as well as protein post-translational modification [306, 307].

Physical host tissue injury during parasite development or feeding, along with an active wound-healing mechanism, leads to enhanced cell transformation and proliferation of cancer cells [309]. This is particularly true in biliary injury caused by liver trematodes feeding activities [31]. Long-lasting cycles of healing and re-injury brought on by the fluke suckers cause cells to be prone to accumulate additional lesions and build up strong proliferative responses via dysregulated signaling pathways [307, 309].

Secondly, oxidative stress induced during inflammation by reactive oxygen species (ROS) and reactive nitrogen species (RNS) is a key factor in parasite-related cancerogenesis. Parasitic noxae summon macrophages, neutrophils, and eosinophils, that produce reactive compounds such as nitric oxide (NO), and superoxide radical (O_2_^−^) [308, 310], that can lead to DNA damage directly, or indirectly by lipid peroxidation, reactive aldehyde production, like 4-hydroxy-2-nonenal (HNE), cyclooxygenase-2 (COX-2) activation, and 8-oxo-7.8-dihydro-2′-deoxyguanosine (8-oxodG) release. The latter has been used as a biomarker for DNA oxidative damage in a number of parasite-related malignancies [32, 311].

Thirdly, inflammatory mediators can concur to the dysregulation of several signaling pathways, including p53, Jak/Stat, retinoblastoma protein (RB), and NF-κB [32, 312], with mechanical and free-radical-mediated injuries. Many oncogenes, like p53, SMAD4, RB, EGFR, and ERBB2, as well as altered DNA methylation and transcriptional profiles, are implicated in parasite-induced malignancy development [6, 32, 312–315]. By increasing the expression of tumor-promoting cytokines, the activation of the transcription factor NF-κB links inflammation to cancer [312]. Other molecular processes at play include aberrant stimulation of the Wnt/-catenin, PI3K/AKT/mTORC1 pathways and downregulation of p53, RB1, and p16INK4 expression [306, 314]. Higher levels of pro-inflammatory cytokines, including tumor necrosis factor-alpha (TNF-α), platelet-derived growth factor (PDGF), vascular endothelial growth factor (VEGF), transforming growth factor-beta (TGF-β), and IL-1, IL-6, and IL-8, enhance angiogenesis, metastatic dissemination, and cell proliferation [7, 22, 117, 279]. Accordingly, dogs with cancer brought on by spirocercosis have considerably higher levels of IL-8, VEGF, and FGF expression, compared to non-neoplastic controls [316].

### Parasite excretory and secretory products (ESPs)

The development of cancer induced by parasites is not solely caused by chronic inflammation, as there are several other mechanisms at play. One such mechanism is the release of excretory and secretory products (ESPs) by the parasite, which are molecules that interact with the host and modulate the parasite-host interface. ESPs can be released actively through the secretion of functional products or passively through the excretion of waste products [317]. The role of ESPs in parasite-induced cancer has been extensively reviewed recently [22], and a variety of pro-inflammatory, oxidative, and genotoxic biomolecules, growth factors, and proteins involved in parasite feeding activity, tissue invasion, immunomodulation, and cell proliferation have been identified [22, 317]. The complex interplay between ESPs and host cells is believed to accelerate and regulate the endogenous parasite-related inflammatory process. Since ESPs are products of coevolutionary processes in which parasites have had to survive in hostile environments for extended periods of time, it is not surprising that their effects may differ between host species and individuals [31]. From a non-evolutionary perspective, the different carcinogenic potential of similar parasitic agents (eg. *Opistorchis viverrini* vs *O. felineus*) [62] and the resistance of some host species to tumor development would have remained unanswered questions [318].

The impact of ESPs on host cellular homeostasis, which can contribute to malignant transformation, has been studied in Spirocercosis [22], Trichinellosis [167], Fascioliasis [218], Opistorchiasis [319], Clonorchiasis [320], Schistosomiasis [321], Strobilocercosis [239], and Theileriosis [279]. Certain compounds, such as the ferritin heavy chain protein (FHC) generated by *C. sinensis*, can increase the formation of ROS and RNS, as well as of endogenous proinflammatory cytokines [320]. Additionally, *Schistosoma* can directly release nitrosamines, free radicals, beta-glucuronidase, and COX-2 enzymes [8, 322]. Together with other fluke-derived metabolites, like oxysterols and catechol estrogens, ESPs increase oxidative stress, along with neoplastic risk [206, 319]. NF-κB and TNF-α may also be overexpressed directly under the action of other *C. sinensis* ESPs via Toll-like receptor stimulation [323], causing a time-dependent rise in proinflammatory cytokines (IL-1 β, IL-6, and TNF-α), and some anti-inflammatory cytokines (IL-10, TGF-1, and TGF-2) [324]. Other helminth proteins cause abnormalities that are typical of malignancies, such as the inhibition of apoptosis and epithelial-mesenchymal transition (EMT) [7, 22, 167].

Thioredoxin (TRX), thioredoxin peroxidase (TPX), and granulin are examples of direct cell mitogens. The granulin-like growth factor (GRN-1) released by *O. viverrini* or *C. sinensis* accelerates wound healing, angiogenesis, tumor invasion, and metastatic spread, whereas TRX and TPX can suppress apoptosis [6, 31, 325]. Additionally, the Interleukin-4-inducing principle of *Schistosoma mansoni* eggs (IPSE), an immunomodulatory protein produced by *Schistosoma* genus eggs, was found to play a role in promoting endothelial and urothelial proliferation [326]. Flukes may expand their food source or enhance their egg expulsion by promoting cell proliferation, and carcinogenesis may be an unintentional result of this route [31, 326].

The depicted scenario is a very dynamic research area. Despite the recent huge advances in proteome analysis, the precise mechanisms by which some ESPs induce cancer while others do not are still unclear, likewise their evolutionary implications [318, 327]. Thus, cancer-causing parasites other than the well-known human flukes, as long as non-cancerogenic parasites that still induce severe inflammatory responses or massive proliferative changes (e.g. trichostrongylids or coccidia), need to be further investigated [22, 328, 329].

### Immunomodulation

In order to survive in the host, parasites modulate a variety of immunologic processes [330]. A shift in the Th1-Th2 balance towards a Th2 response is a common outcome of most helminth infections in humans and animals, which inhibits Th1 cell-mediated immunity involved in parasite clearance, but also in tumor immunosurveillance and rejection [30, 167, 330–332]. Chronic infections sustain the increased production of Th2-associated cytokines (IL-4, IL-5, IL-9, IL-10, and IL-13), perpetuating the inhibition of Th1 responses [30, 330]. Interleukin-4-inducing principle (IPSE), a protein released by *Schistosoma*, activates basophils and mast cells to secrete IL-4 and IL-13, especially during *S. mansoni* eggs deposition [6, 326, 333]. The Th1/Th2 regulatory gene suppressor of cytokine signaling 5 (SOCS5) and interferon-gamma (IFN-γ) have been demonstrated to be significantly modified by *O. felineus* [334]. In addition, immunomodulatory substances, such as galectin found among *S. lupi*-ESPs, may also aid the immunoediting process [335]. Consequently, the growth of Treg cells and alternative activation of M2-polarized macrophages brings along an increase in parasite-host tolerance, tissue repair, and suppression of antitumor immune responses [330, 332, 336].

### Increased susceptibility to environmental carcinogens

In recent years, researchers have studied an indirect carcinogenic process, particularly through experimental trematode infections in animal models [6]. This process may result in reduced clearance of food or environmental carcinogenic substances (such as nitrosamines, aromatic amines, and aflatoxins) due to mechanical damage, a persistently inflammatory environment, and release of parasite ESPs, which can lead to key metabolic hepatic enzymes becoming less active [21, 30].

Experimental infections with liver (*Opistorchis* spp., *C. sinensis*), and blood (*Schistosoma* spp.) flukes have been found to cause inflammatory changes in the absence of carcinogens [6, 337]. However, the development of neoplasia required treatment with low doses of nitrosamines (which are not cancerogenic by themselves) [6, 7, 31, 308, 337]. In fact, when hamsters’ bile ducts were surgically tied to mimic the damage caused by fluke infections, sub-carcinogenic oral doses of nitrosamines were demonstrated to cause biliary tumor development [338].

Older theories attributed cancer initiation to nitrosamines, with parasite infection providing the proliferative stimulus to start cell promotion [339]. However, liver fluke infection can also enhance nitrosation of amine precursors. *Opistorchis* and *Fasciola* infections have been linked to induction of the CYP2A5 enzyme, an isoform of cytochrome P-450 (CYP), which participates in metabolic activation of carcinogens, such as N-dimethylnitrosamine (NDMA) and aflatoxin B1 (AFB1), with high levels of NDMA found in the bile duct in humans and hamsters [308, 340, 341]. *Schistosoma mansoni* has also been experimentally linked to metabolic activation of procarcinogens, with a rise of 300% in aflatoxin metabolites [342]. These findings suggest that parasites play a role in amplifying the effects of environmental carcinogens, thereby indirectly raising the risk of cancer.

### Synergic parasite-virus coinfections

Cancerogenesis is a multistep and multifactorial process, and coinfection with two or more pathogens has been linked to higher cancer risk than infection with either pathogen alone [343]. For instance, many studies have shown the synergistic effects of EBV, and the haemosporidian protozoan *Plasmodium falciparum* (a causal agent of malaria) in the development of Burkitt’s lymphoma (BL) [344]. BL, the most common childhood cancer in Africa, is an aggressive B-cell malignancy and has a higher incidence where malaria is endemic [345]. The mechanisms involved in cancerogenesis include the cysteine-rich interdomain region 1α (CIDR1α)-mediated expansion of EBV-infected B-cell populations, along with B-cell activation-induced cytidine deaminase (AID)-related *c-myc* and *IgH* translocations, discussed in detail elsewhere [343, 346, 347]. Even though malaria has limited veterinary implications, the discovery that the zoonotic simian protozoa, *P*. *knowlesi,* had been misdiagnosed as *P. malaria* for years [348], and the fact that other unknown *Plasmodium* sp. lineages may be largely overlooked in human medicine [349], suggest that further research is warranted to explore the potential cancerogenic role of other simian and avian forms of malaria [350].

*Strongyloides stercoralis* causes a chronic parasitic infection in humans and animals known as strongyloidosis, which is negatively influenced by co-infection with human T-cell leukemia/lymphoma virus type 1 (HTLV-1) [351, 352]. In fact, the odds for disseminated strongyloidiasis and hyperinfection syndrome are higher in HTLV-1 co-infected patients [351]. However, co-infection may also have a detrimental influence on the outcome of HTLV-1 infection [353]. Adult T-cell Leukemia/Lymphoma (ATLL) is the most severe manifestation of HTLV-1 infection, and S. stercoralis may be involved in the oncogenesis as suggested by increased HTLV-1 proviral loads and the earlier onset of ATLL in *S. stercoralis*-positive carriers [353, 354]. In particular, the parasitic infection may have a role in promoting HTLV-1-infected cell proliferation and immune-related genes expression, which may play a significant role in the development of ATLL [352].

Liver sarcoma induced by *T. taeniformis* [244, 247] and *Ollulanus*-related stomach cancer have also been hypothesized to arise in patients coinfected by viruses [144]. There is evidence to suggest that both *S. mansoni* and *C. sinensis* may indirectly potentiate the development of hepatocellular carcinoma by HBV and HCV [189, 215]. Lastly, the association between *T. annulata* and BLV-induced lymphoma in calves described by Al-Dubaib et al. [288] may be explained by *Theileria*-induced lymphoid proliferation boosting neoplastic transformation induced by the retrovirus.

### Parasite-Microbiota interactions

The role of commensal bacteria in cancer development has gained momentum in recent years, leading to a surge of research on the topic [355]. A growing body of evidence suggests that the gut, oral, and vaginal microbiomes may influence cancer development through various mechanisms [356, 357].

The composition of the host's microbiota is significantly shaped by several parasites [358, 359]. Therefore, the link between parasite infection and cancer may depend on the host's microbiome composition at the time of infection, making it even more challenging to prove [360]. For example, *Trichomonas vaginalis* releases ESPs that cause a shift in the vaginal microbiota from lactobacilli to human bacterial vaginosis causative agents, including the cancer-associated microorganism *Chlamydia trachomatis* [361].

*Schistosoma* species also promote microbiome variations, favoring communities of nitrate-reducing bacteria that, in turn, form nitrosamines [360, 362, 363]. Furthermore, *O. viverrini* may introduce the carcinogenic bacterium *Helicobacter pylori* and other bacteria into the biliary tree [364, 365].

The fact that bacteria are present within or associated with most cancer-causing parasites has not received sufficient attention [244, 360]. Further research on the relationship between microbiota, parasites, and cancer will undoubtedly be crucial in clarifying the infectious causes of cancer [359].

### Hijacking host-cell metabolism

The neoplastic transformation of bovine leukocytic cells induced by *Theileria* represents a distict mechanism involved in parasite-driven cancerogenesis. *Theileria* has evolved complex strategies to interact with host cell metabolic pathways and exploit their genetic and epigenetic machinery to alter host cell phenotype to a cancer-like one [279, 284, 291]. Living freely in the host cytoplasm, it secretes ESPs that act directly as "epigenators" [366]. These reversible signals trigger epigenetic initiators that interact with chromatin and its transcription [290, 366]. Another hallmark of *Theileria*-induced transformation is the production of the "Warburg effect" metabolic signature, which shifts host metabolism from oxidative phosphorylation to aerobic glycolysis [35, 284, 367].

During *Theileria*-induced host cell transformation, the anti-apoptotic c-Jun N-terminal kinase (JNK) and host nuclear factors c-Myc, NF-κB, and AP-1 are among the signaling pathways affected [28, 279, 368]. Moreover, *Theileria* alters host cell kinematics, enhances motility, and causes infected host cells to behave as leukocyte metastases [27, 369].

The parasite homolog of Phosphorylation-Dependent Peptidyl-Prolyl Cis/Trans Isomerase PIN1 (TaPIN1) [284, 370] is a major epigenator expressed by transforming *Theileria* spp., but not by non-transforming or closely related apicomplexan parasitic species. The TaPin1 protein interacts with two of the main host signaling pathways involved in proliferation (via Fbw7 ubiquitin ligase, c-Jun transcription factor, and onco-miR-155 overexpression) [370, 371], and metabolic homeostasis (via PKM2 through HIF1α transcription factor) [284]. Alongside D-2-hydroxyglutarate (D-2-HG), which inhibits histone lysine demethylases (KDMs) and the DNA methylating Ten-Eleven Translocation (TET) enzyme family [279, 372], it represents the likeliest inducer of tumor-like phenotype.

### Cancer arising in parasites

A 2015 report by Muehlenbachs and colleagues [373] describes a peculiar interaction between host and parasite during cancerogenesis. An HIV-positive patient infected by dwarf tapeworm (*Hymenolepis nana*) presented a metastatic neoplasm of unknown origin. A non-human origin was postulated by neoplastic cell morphology. Further immunohistochemical and molecular analysis confirmed that malignant cells arose from the worm *H. nana* and invaded the host’s tissues [373], indicating that a carcinogenic mechanism may also occur in the parasite itself. In this respect, it has been proposed that the absence of typical host-defense signals (due to immunodeficiency) can lead to aberrant tapeworm growth, tissue spread, and ultimately neoplasia in the parasite as well [374]. This phenomenon may be widespread in human and veterinary medicine [375]. Numerous reports of aberrant transformation among cestodes that infect humans and other animals, including *Mesocestoides* [376], *Versteria* [377, 378], and *Spirometra* species [379–381] have been published. However, caution is needed when distinguishing a true neoplastic phenomenon from parasite life cycle stages based on abnormal larval proliferation, as in the enigmatic case of the fatal zoonotic tapeworm *Sparganum proliferum* [375, 382]. From a broader perspective, this scenario has led to the theorization of new and unconventional interactions between host and parasite [299].

Cancer in non-human hosts has a wide spectrum of biological features and veterinary oncology is familiar with atypical routes of tumor transmission, such as transmissible infectious cancers in dogs, Tasmanian devils, golden hamsters, and marine bivalves [25]. Thus, research in the veterinary field could play a significant role in investigating this phenomenon.

## Antitumor activity

So far, we have explored the role of parasitic diseases as cancer inducers or promoters. However, in recent years, 13 parasitic agents, some of which are familiar to veterinarians (such as *Toxoplasma gondii*, *Echinococcus granulosus*, *Trichinella spiralis*, and *Toxocara canis*), have been investigated as mediators of anti-tumor responses, further complicating an already complex picture [383]. Several anti-tumor mechanisms, including the sharing of common antigens and the enhancement of active tumor surveillance via antibody-dependent cellular cytotoxicity (ADCC), have been proposed and detailed extensively elsewhere [384–386]. A comprehensive insight into these mechanisms is beyond the scope of this paper. However, it cannot be denied that this paradoxical dual-role of parasites emphasizes even more how much the host-parasite interaction affects the etiopathogenesis of cancer and how research into this relationship can benefit both prevention and treatment.

## Conclusive remarks

In this paper, the cancerogenic roles of 15 helminths, one arthropod, and two protozoa of veterinary interest were discussed through an extensive literature review. We included several wild and domestic animal species, along with different neoplastic histotypes, such as carcinomas, sarcomas, melanomas, lymphoma, and epithelial and mesenchymal benign tumors. We covered a large span of research from different historical periods, starting from the story of the first hypothetical cancer-causing parasite to recent *Theileria* spp. molecular investigations. In the last part of the paper, a veterinary-oriented overview of the cancerogenic mechanisms involved in the parasite-host dialogue was provided. The link between chronic inflammation and cancer is a well-known phenomenon, but parasites can also encourage additional, even bizarre mechanisms to further promote malignancy. Given the heterogeneity of the various types of tumors and parasite species, there is expected to be variability in pathogenetic mechanisms. The important role played by the host species and strain-related or individual factors in the interaction with parasites emerges transversally. Various strains of the same parasite could bring along variations in host preference and pathogenicity, as with *O. viverrini*.

Another essential issue related to the development of cancer after long post-exposure intervals is the lower average age of many domesticated species compared to humans. This aspect may help explain the shortage of veterinary reports, which could be hiding the true cancerogenic potential of some parasitic agents.

Drawing conclusions on the association between cancer and some of the parasite infections listed in the first part of this review is challenging, and our knowledge will remain incomplete until reproducible epidemiologic and experimental data are available. However, the aim of the paper was to shed light on the current knowledge in the field from a veterinary perspective, since a comprehensive and updated work in such a dynamic scenario was lacking. We hope that this paper can help further investigations on cancer-causing parasites in veterinary medicine and stress their importance as useful spontaneous animal models in a One Health perspective.

## Data Availability

Not applicable.
